# DNA Demethylase ROS1 Interferes with DNA Methylation and Activates Stress Response Genes in Plants Infected with Beet Severe Curly Top Virus

**DOI:** 10.3390/ijms26062807

**Published:** 2025-03-20

**Authors:** Taicheng Jin, Yushuo Li, Xu Sun, Yidi Li, Zhuyi Xiao, Weiyan Wang, Jiaxue Yu, Liping Yang

**Affiliations:** The School of Life Sciences, Jilin Normal University, Siping 136000, China; jintaicheng8128@163.com (T.J.); m15568356195@163.com (Y.L.); sunxu1206@163.com (X.S.); lyd9999a@163.com (Y.L.); 13086887230@163.com (Z.X.); wwy98818@163.com (W.W.); 15330658182@163.com (J.Y.)

**Keywords:** ROS1, DNA demethylation, stress response genes, BSCTV

## Abstract

DNA methylation is one mechanism of epigenetic regulation in plants. Small interfering RNAs (siRNAs) targeted endogenous genes and caused the promoters to be hypermethylated, namely RNA-directed DNA methylation (RdDM). Repressor of silencing 1 (ROS1) is an active DNA demethylase involved in the regulation of DNA methylation. This study indicates that ROS1-mediated DNA demethylation plays important roles in regulating the expression of these stress response genes and in response to biotic stresses. Further experiments confirmed that the expression level of the *ROS1* gene was significantly upregulated in *A. thaliana* plants infected with *beet severe curly top virus* (BSCTV). Moreover, the DNA sequencing results demonstrated that ROS1 interferes with DNA methylation of repeat regions in the promoters of *ACD6*, *GSTF14*, and *ACO3* in *A. thaliana* plants infected with BSCTV. These findings reveal the epigenetic mechanisms by which ROS1 regulates the expression of the stress response genes, thereby improving the adaptability of plants to biotic stresses.

## 1. Introduction

RNA silencing has been an important defense pathway in plants over the course of evolution [1,2,3]. The RNA-induced silencing complex (RISC) recognizes and cleaves target mRNAs, resulting in post-transcriptional gene silencing (PTGS) [4,5,6]. In plants, the RNA-directed DNA methylation (RdDM) pathway targets repeat sequences in the promoter of endogenous genes or invasive DNA sequences, which leads to transcriptional gene silencing (TGS) [7]. The RdDM pathway can be triggered by the transcription of inverted repeats or dispersed repeats, and heterochromartin-realted siRNAs play an important role in DNA methylation [8,9]. In the process, the plant-specific Dicer-like ribonuclease 3 (DCL3) cleaves dsRNA into 24 nt siRNAs, which are loaded in Argonaut 4 (AGO4). AGO4 protein recruits methyltransferase DDM1, MET1, and CMT3 to form RISC and perform the de novo DNA methylation of cytosines and maintenance of methylation in the symmetric CG and CHG contexts (H is A, C, or T) and the asymmetric CHH context [10,11,12,13,14]. The RdDM pathway also plays an important role in defense against invasive DNA sequences [9].

Plant viruses counter DNA methylation-mediated defense by the virial suppressors, including DNA viruses and RNA viruses [15,16,17]. A previous study revealed that the C2 protein encoded by BSCTV counters host defense responses via the regulation of S-adenosylmethionine decarboxylase 1 (SAMDC1), which increases the dcSAM/SAM ratio and inhibits DNA methylation [18]. Our previous studies revealed that the viral suppressor C2 can decrease the DNA methylation of dispersed repeats (DRs) in the promoter of *ACD6*, which is an upstream regulator gene in the salicylic acid (SA) pathway in *Arabidopsis thaliana* [7]. We also found that the viral suppressor HC-Pro from *tobacco vein banding mosaic virus* (TVBMV) decreases in DNA methylation in the promoter and activates *Accelerated cell death 6* (*ACD6*) and *NONEXPRESSOR OF PR GENES 1* (*NPR1*) involved in the SA pathway and auxin biosynthesis genes [15,19].

Active DNA demethylation is performed by DNA demethylases in *A. thaliana* plants, including the repressor of silencing 1 (ROS1), 5-mC glycosylase (Demeter, DME), Demeter-like protein 2 (DML2), and Demeter-like protein 3 (DML3) [20,21]. In plants, ROS1 regulates DNA methylation and promotes the expression of defense-related genes [22,23]. Studies have shown that *ros1* mutants are more susceptible to *Pseudomonas syringae* infection [24]. ROS1 has important functions in the regulation of stress tolerance and pathogen defense in plants [25,26,27]. For example, ROS1 promotes the expression of NICOTINAMIDASE 3 (NIC3) and participates in the abscisic acid (ABA) response in *A. thaliana* plants [28]. Our previous studies indicate that ROS1-mediated DNA demethylation plays an important role in the transcriptional activation of defense-related genes and stress response genes when *A. thaliana* plants are subjected to abiotic stresses [29]. However, whether ROS1-mediated DNA demethylation plays a role in virus–host interactions still remains unclear. And the molecular mechanisms that ROS1 actively in response to biotic stresses remain unknown.

In this study, we found that *ros1*-defective mutants are hypersensitive to BSCTV infection and that the accumulation of BSCTV in virus-infected *ros1* mutants was clearly greater than that in virus-infected Col-0 plants. These findings revealed the important role of ROS1 in regulating the expression of the stress response genes, including ABA pathway-related gene *ACO3*, stress-responsive gene *GSTF14,* and defense-related gene *ACD6*. Further experiments demonstrated that *ROS1* was induced by BSCTV in *A. thaliana* plants and involved in regulating DNA demethylation in the promoters of *ACD6*, *GSTF14*, and *ACO3*. This study suggests that ROS1 actively responds to biotic stresses by regulating the DNA methylation of stress response genes, thereby improving the adaptability of plants to the environment.

## 2. Results

### 2.1. Symptoms of BSCTV-Infected Arabidopsis Thaliana Col-0 and ros1 Plants

**Figure 1:**
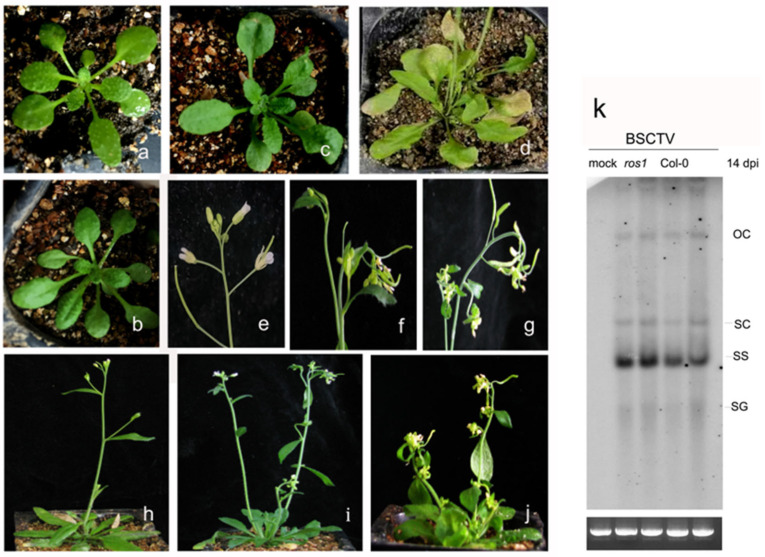
BSCTV symptoms in *Arabidopsis* Col-0 plants and *ros1* mutants. (**a**) *Arabidopsis thaliana* Col-0 plants were used as controls. (**b**) *Arabidopsis thaliana ros1* mutants presented no obvious changes. (**c**) The leaves of BSCTV-infected Col-0 exhibited deformation at 7 dpi. (**d**) The leaves of BSCTV-infected *ros1* mutants presented more severe deformation than BSCTV-infected Col-0 at 7 dpi. (**e**) Mock-infected *ros1* mutants presented no obvious changes. (**h**) Mock-infected Col-0 plants were used as controls. (**f**,**i**) BSCTV-infected Col-0 presented curled-top symptoms at 14 dpi. (**g**,**j**) *ros1* mutants presented more severe curved-top symptoms than BSCTV-infected Col-0 at 14 dpi. (**k**) The accumulation of BSCTV in virus-infected Col-0 and *ros1* plants was determined by Southern blotting at 14 dpi, and the accumulation of virus in mock-infected plants served as a control.

*A. thaliana* wild-type Col-0 plants and *ros1*-defective mutants were used as plant materials, and *Agrobacterium* inoculation was used to perform BSCTV infection. The mock-infected Col-0 plants used as controls (Figure 1a) and the leaves of the mock-infected *ros1* mutants presented no developmental defects (Figure 1b), and the leaves of the BSCTV-infected *ros1* mutants presented more severe curling deformation and yellowing (Figure 1d) than those of BSCTV-infected Col-0 plants (Figure 1c) at 7 days post-inoculation (dpi). The most typical viral symptom was a curled-top phenomenon in BSCTV-infected Col-0 at 14 dpi (Figure 1f,i). The flowers of BSCTV-infected *ros1* mutants developed more severe curled-top symptoms (Figure 1g,j), whereas the flowers of the mock-infected *ros1* mutants presented no developmental defects (Figure 1e).

Our previous study revealed that *ros1* mutants presented more severe phenotypic changes in p-HCPro-infected Col-0 plants [19]. To examine whether *ros1* mutants are more susceptible to BSCTV infection, the accumulation of BSCTV in virus-infected Col-0 and *ros1* plants was determined by Southern blotting at 14 dpi. Consistent with these observations, the Southern blotting results indicated that the accumulation of BSCTV in virus-infected *ros1* mutants was clearly greater than that in virus-infected Col-0 plants (Figure 1k). These results suggest that ROS1 might play a role in the defense against the invasion of viruses.

### 2.2. Increased Expression of Stress Response Genes Is Mediated by C2 in BSCTV-Infected and C2 Transgenic Plants

**Figure 2:**
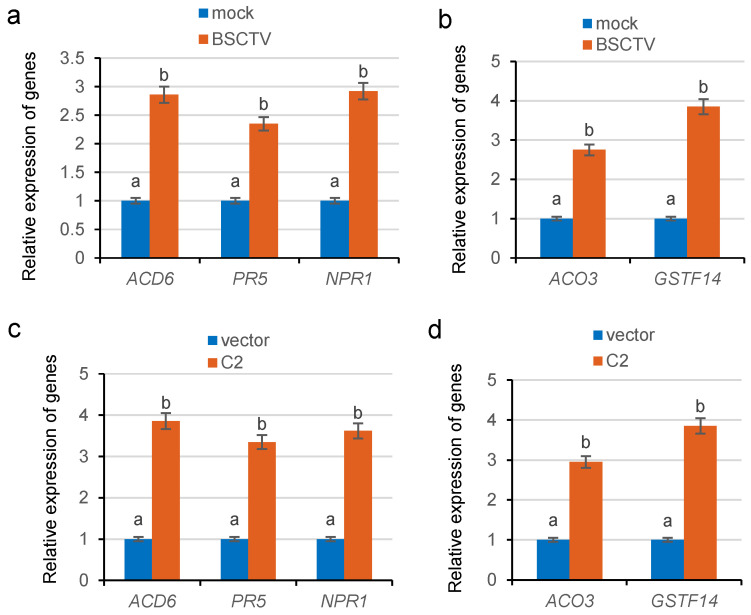
Analysis of gene expression levels in BSCTV-infected plants and C2 transgenic plants. (**a**,**b**) RT-qPCR analysis of the expression levels of the stress response genes *ACD6*, *GSTF14,* and *ACO3* and the defense-related genes *NPR1* and *PR5* in BSCTV-infected Col-0. (**c**,**d**) RT-qPCR analysis of the expression levels of the stress response genes and the defense-related genes in C2 transgenic plants. Means identified by different letters are significantly different from each other. Error bars represent SEs from three biological replicates. One-way ANOVA followed by Tukey’s multiple comparison test was used for statistical analysis (*p* < 0.05).

Our previous study revealed that *tobacco vein banding mosaic virus* (TVBMV) increased the expression of defense-related gene *ACD6* [7]. To examine whether BSCTV infection also increases the expression of stress response genes *GSTF14* and *ACO3*, total RNA was extracted from mock-infected and BSCTV-infected Col-0 plants at 14 dpi. The RNA was used for RT experiments to obtain cDNA, and the expression of stress response genes was determined by qPCR. When the expression levels in mock-infected Col-0 plants were used as a control, the expression levels of *ACD6*, *GSTF14,* and *ACO3*, the defense-related genes *NPR1,* and *pathogenesis-related gene 5* (*PR5*) were significantly increased in BSCTV-infected plants (Figure 2a,b).

To confirm whether the increased expression of these genes was also regulated by the virial suppressor C2 encoded by BSCTV, the expression levels of these genes in C2 transgenic plants were determined via RT-qPCR. The results confirmed that the expression levels of stress response genes and the defense-related genes were significantly upregulated in C2 transgenic plants compared with those in the empty-vector controls (Figure 2c,d). These results indicated that the BSCTV and C2 proteins can induce the expression of stress response genes.

### 2.3. The Repressor of Silencing ROS1 Is Involved in Regulating the Expression of the Stress Response Genes in BSCTV-Infected Plants

**Figure 3:**
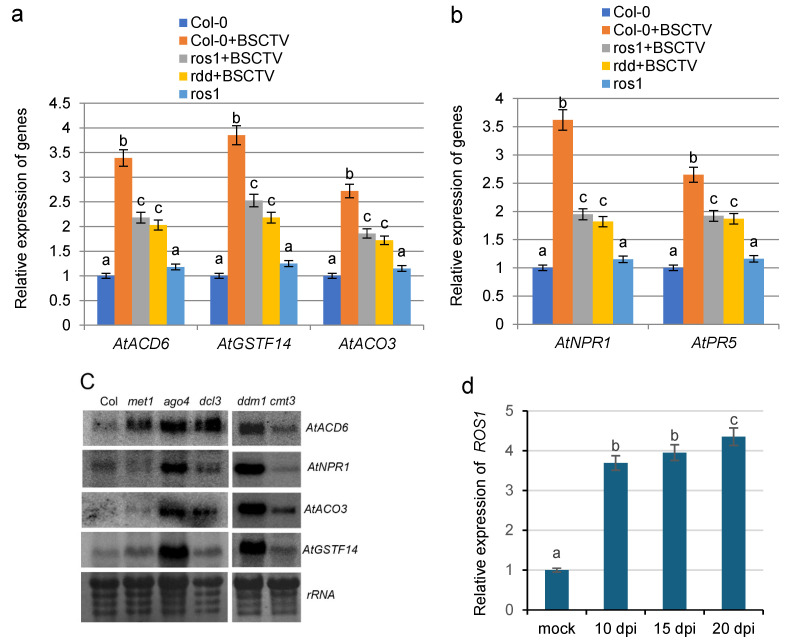
Analysis of gene expression levels in Col-0 and *ros1* plants infected with BSCTV. (**a**) Expression in mock-infected plants was used as a control, and RT-qPCR analysis of the expression levels of stress response genes in BSCTV-infected Col-0, *ros1*, and *rdd* mutants was performed. (**b**) RT-qPCR analysis of the expression levels of the defense-related genes in BSCTV-infected Col-*0, ros1*, and *rdd* plants. (**c**) Northern blotting analyses of the expression levels in the mutants *ago*4, *met*1, *dcl*3, *ddm*1, and *cmt*3. (**d**) RT-qPCR analysis of the expression levels of *ROS1* in BSCTV-infected Col-0; *GAPDH* served as a reference gene. Means identified by different letters are significantly different from each other. The error bars represent the SEs from three biological replicates. One-way ANOVA followed by Tukey’s multiple comparison test was used for statistical analysis (*p* < 5).

Our previous studies revealed that ROS1 plays an important role in the regulation of the stress response genes in *A. thaliana* plants subjected to cold stress [29]. To determine whether ROS1 is involved in the regulation of gene expression in BSCTV-infected plants, the expression levels of the stress response genes were analyzed via RT-qPCR in BSCTV-infected Col-0 plants and *ros1* mutants. Relative to the expression levels in the mock-infected Col-0 plants used as controls, the expression of *ACD6*, *GSTF14*, and *ACO3* was significantly increased in BSCTV-infected Col-0 plants at 14 dpi. However, this increase in expression in the *ros1* mutants at 14 dpi was partially inhibited (Figure 3a). The expression levels of these genes in the *ros1* mutants were not significantly altered compared with those in mock-infected Col-0 plants. Moreover, the results demonstrated that ROS1 also plays a role in the regulation of defense-related genes *NPR1* and *PR5* in BSCTV-infected plants (Figure 3b).

We detected the expression of related genes in plants mutated at key functional elements of the RdDM pathway. Compared with those in Col-0 ecotypes, the expression levels of *ACD6*, *NPR1*, *ACO3,* and *GSTF14* clearly increased in the mutants *ago4*, *met1*, *dcl3*, *ddm1*, and *cmt3* (Figure 3c). In addition, the results revealed that the expression level of *ROS1* was significantly greater in BSCTV-infected plants than in mock-infected plants at 10 dpi, 15 dpi, and 20 dpi (Figure 3d). Our previous studies revealed that HCPro, which is encoded by TVBMV, induced the expression of *ROS1* [19]. These results indicate that both the repressor of silencing ROS1 and the virial suppressor C2 are involved in the activation of the stress response genes and the defense-related genes.

### 2.4. Increased Gene Expression Is Correlated with ROS1-Mediated DNA Demethylation in the Promoters of Stress Response Genes

**Figure 4:**
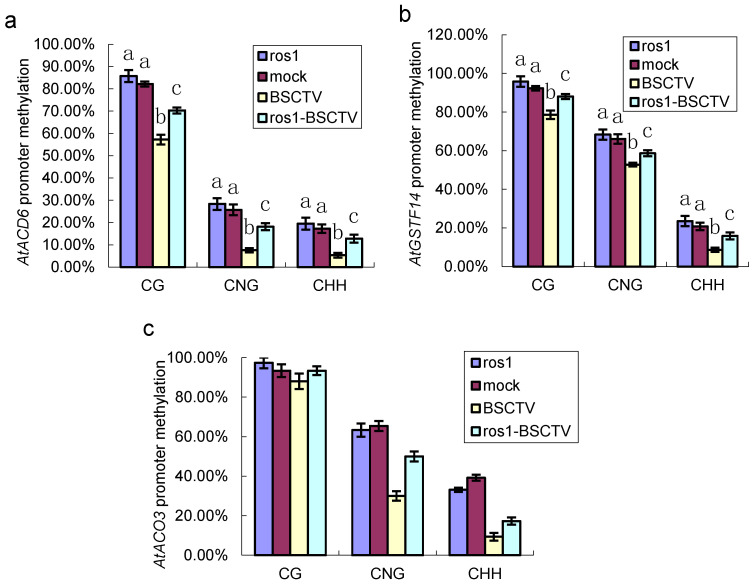
DNA methylation analysis of promoters and analysis of gene expression in the mutants. (**a**–**c**) DNA methylation analysis at CG, CNG, and CHH sites in the promoters of *ACD6*, *GSTF14*, and *ACO3*. The methylation levels in the mock-infected plants were used as controls. ros1-BSCTV represents the methylation levels in the BSCTV-infected *ros1* mutants. Means identified by different letters are significantly different from each other. The error bars represent the SEs from three biological replicates. One-way ANOVA followed by Tukey’s multiple comparison test was used for statistical analysis (*p* < 5).

To investigate whether increased gene expression is correlated with ROS1-mediated DNA demethylation, we performed sequencing analysis of the DNA methylation of repeat sequences at the promoters of related genes in BSCTV-infected Col-0 and *ros1* plants. The sequencing analysis discovered that the DNA methylation levels in the *ACD6* promoter were reduced by 24.91% (a change from 82.16 to 57.25%) in CG sites, by 18.09% (a change from 25.65 to 7.56%) in CHG sites, and by 11.93% (a change from 17.28 to 5.35%) in CHH sites in BSCTV-infected Col-0 compared with those in mock-infected Col-0 (Figure 4a). The DNA methylation levels in the *ACD6* promoter were reduced by 15.50% (a change from 85.75 to 70.25%) in CG sites, by 10.22% (a change from 28.33 to 18.11%) in CHG sites, and by 6.72% (a change from 19.52 to 12.80%) in CHH sites in BSCTV-infected *ros1* plants compared with those in *ros1* mutants. The results indicate that the decrease in DNA methylation in the *ACD6* promoter in BSCTV-infected *ros1* mutants was partially inhibited (Figure 4a).

Sequencing analysis of DNA methylation in the promoters of *AtGSTF14* and *AtACO3* further confirmed the role of ROS1. Compared with those in mock-infected Col-0, the DNA methylation levels in the promoters of *GSTF14* were reduced by 13.74% (a change from 92.30 to 78.56%) in CG sites, by 14.29% (a change from 66.04 to 52.75%) in CHG sites, and by 12.13% (a change from 20.78 to 8.65%) in CHH sites in BSCTV-infected Col-0, whereas the decreases in DNA methylation in the promoter of *GSTF14* in the BSCTV-infected *ros1* mutants were clearly inhibited (Figure 4b). The DNA methylation levels in the promoters of *ACO3* were reduced by 5.33% (a change from 93.77 to 88.00%) in CG sites, by 35.43% (a change from 65.43 to 30.00%) in CHG sites, and by 31.03% (a change from 39.19 to 9.38%) in CHH sites in BSCTV-infected Col-0 plants, whereas the decreases in the DNA methylation in the promoter of *ACO3* in the BSCTV-infected *ros1* mutants were clearly inhibited (Figure 4c). Moreover, compared with those in mock-infected Col-0 plants, the DNA methylation levels in the promoters of *ACD6*, *GSTF14*, and *ACO3* in the *ros1* plants were clearly not altered (Figure 4a–c). These results demonstrated that the repressor of silencing ROS1 is involved in the process of DNA demethylation in the promoters of these genes.

## 3. Discussion

### 3.1. ROS1 Is Involved in the Regulation of Stress Response Gene Expression

Plant virus infection can activate a number of signaling pathways, including the salicylic acid (SA) pathway and jasmonic acid (JA) [7,15]. Gene chip analysis of *Arabidopsis thaliana* infected with *Cabbage leaf curl virus* (CaLCuV) found that a total of 5365 genes showed differential expression after virus infection, and data analysis showed that the virus activated the SA pathway-mediated immune response and affected the gene expression of multiple signaling pathways [30]. Glutathione S-transferases (GSTs) respond to biotic and abiotic stresses [31,32,33]. The activity of GSTs is closely related to the stress tolerance of plants [34]. *GSTF14* is an endogenous gene of the GST superfamily. *ACO3* is an ABA pathway-related gene, and *ACD6* is a defense-related gene. Our previous studies indicate that the stress response genes *ACD6*, *GSTF14*, and *ACO3* are RdDM pathway target genes, and the RdDM pathway plays an important role in maintaining the low transcription levels of these endogenous genes in Col-0 plants [29].

ROS1 reduces the methylation of immune-related genes and induces their expression to complete the defense response [23,26]. Increased expression of the upstream regulator ACD6 in plants can further induce SA accumulation and the acquisition of systemic resistance [35]. In this study, we discovered that *ros1* mutants are more susceptible to BSCTV infection (Figure 1), and the accumulation of the virus in BSCTV-infected *ros1* mutants was increased compared with that in BSCTV-infected Col-0 plants (Figure 1k). The findings suggest that ROS1 might play a role in the defense against viruses. Our results revealed that BSCTV C2 increased in the expression of stress response genes *ACD6*, *GSTF14*, and *ACO3* and defense-related genes *NPR1* and *PR5* (Figure 2). Furthermore, we found the important role of ROS1 in regulating the expression of these genes in BSCTV-infected Col-0 plants (Figure 3a,b). These findings indicate that both the repressor of silencing ROS1 and the virial suppressor C2 are involved in the activation of the stress response genes and defense-related genes. An increase in the expression of stress response genes can further increase the adaptability of plants to biotic stresses.

### 3.2. DNA Demethylation in the Promoters of Stress Response Genes in BSCTV-Infected Plants Is Partially Dependent on ROS1

DNA methylation is an epigenetic marker involved in silencing transposable elements (TEs) in plants and animals [36,37]. The RdDM pathway is a protective mechanism that defends invasive DNA sequences and maintains the stability of the genome [9]. As a counter strategy, viruses effectively block the RdDM pathway and inhibit gene silencing by encoding viral suppressors [15]. Viral suppressor C2 interferes with DNA methylation in the promoters of endogenous genes, which is considered a passive DNA demethylation process [7]. Our previous studies also revealed that the viral suppressor HC-Pro interferes with DNA methylation in the promoters of auxin biosynthesis genes, resulting in an increase in auxin accumulation in plants [19].

The repressor of silencing ROS1 is an active DNA demethylase, which regulates DNA methylation and gene expression in plants [38,39,40]. This study found that the expression of *ROS1* was activated after BSCTV infection and that *ROS1* expression increased over time in *A. thaliana* plants (Figure 3d). Increased *ROS1* expression can increase the DNA demethylation activity of ROS1. A further study revealed that DNA demethylation in the promoters of *ACD6*, *GSTF14*, and *ACO3* in BSCTV-infected Col-0 plants is partially dependent on ROS1 (Figure 4a–c). These results indicate that ROS1 participates in the biotic stress response by regulating the DNA demethylation of these genes, which is an active DNA demethylation process. Our results discover that ROS1 actively in response to biotic stresses by regulating the expression of these stress response genes, thereby promoting plant adaptability to stress.

## 4. Materials and Methods

### 4.1. Plant Growth Conditions

*A. thaliana* ecotype Columbia (Col-0) and *ros1* mutant seeds were used as plant materials for this study. The seeds were surface-sterilized with 30% bleach, washed three times with sterile water, sown on MS plates (pH 5.9), and then incubated at 25 °C with a 16 h light/8 h dark cycle, after which the plants were transferred to soil [7]. The *ros1* and *rdd* [29] mutant seeds were obtained from the Shanghai Center for Plant Stress Biology, Shanghai Institute of Biological Sciences, Chinese Academy of Sciences (CAS).

### 4.2. Agrobacterium Tumefaciens Inoculation

The EHA105 strain carrying pCambia1300-BSCTV was used to infect the plants at an *OD_600_* of 2.0, as described previously, and the inoculation experiments were performed [7]. BSCTV-infected plants that presented obvious viral symptoms were collected and used for DNA or RNA extraction and further experimental analysis. Sixty-four *A. thaliana* ecotype Col-0 plants or *ros1* mutants were infected with BSCTV each time. The BSCTV infection experiments were repeated three times.

### 4.3. Bisulfite Sequencing

Total DNA was extracted at 14 days post-inoculation (dpi) from BSCTV-infected Col-0 and *ros1* mutants using CTAB buffer. The collected DNA was purified using a DNA purification kit. The purified DNA was subjected to bisulfite treatment using an EpiTect bisulfite kit, as described previously [18,29]. The bisulfite-treated DNA was used to amplify from the promoter-specific sequences of *ACD6* (AT4G14400) and *GSTF14* (AT1G49860). The primer pairs used were as follows: *ACD6*-F: 5′-AAGTTTATTGATGAAAGGAG-3′; R: 5′-CTTACTT(G/A)TCTTCATCAA-3′; *GSTF14-*F: 5′-TTTGAAAGTTGGTGTATTAAA-3′; R: 5′-CCCATACCTATCATATTTCAT-3′; *ACO3*-F, 5′- GTAATATTAGTAAAGATGTGT -3′; and *ACO3*-R, 5′-CACTACTTTCATTATACTCTTT-3′. The results of the DNA methylation analysis were obtained from https://www.cymate.org/cymate.html (accessed on 22 December 2024). One-way ANOVA and Tukey’s multiple comparison test were used for statistical analysis, as described previously [29].

### 4.4. RT-qPCR Analysis

Total RNA was extracted from BSCTV-infected Col-0 and *ros1* mutants using TRIzol reagent at 14 dpi. Complementary DNA synthesis was conducted using a reverse transcription kit (Takara, Borri Medical Biotechnology (Beijing) Co., Ltd., Beijing, China) for RT experiments. The DNA was subsequently used for quantitative PCR (qPCR), which was performed using SYBR Green mix (Qiagen, Shanghai BaoKe Biotechnology Co., Ltd., Shanghai, China), as described previously [19]. The *ACD6* (AT4G14400) and *GSTF14* (AT1G49860) probe primer pairs were used as described previously [29].

### 4.5. Southern Blotting Analysis and Northern Blotting Analysis

Total DNA was extracted from the BSCTV-infected Col-0 plants and *ros1* mutants at 14 dpi and used for Southern blotting analysis. The DNA was separated on 1% agarose gels and then transferred to Hybond-Nþ membranes, which were hybridized with DIG-labeled probes [15,19]. For Northern blotting analysis, 10 ug of total RNA of the mutant plants was extracted by using Tizol reagent. The blotting analysis was conducted according to a previously described method [15].

## 5. Conclusions

In summary, an in-depth investigation and elucidation of the molecular mechanisms underlying the interaction between BSCTV and host epigenetic regulation were performed in this study. The results reveal that the transcriptional activation of stress response genes was partially dependent on ROS1 in BSCTV-infected plants. Moreover, a further study demonstrated that ROS1-mediated DNA demethylation plays a role in the regulation of stress response genes, which suggests that ROS1 is involved in the regulation of plant defense mechanisms in response to biotic stresses.

## Data Availability

Data contained within the article.
